# Cushing Syndrome Secondary to Primary Neuroendocrine Lung Carcinoma

**DOI:** 10.1155/2019/1989260

**Published:** 2019-07-24

**Authors:** A. Zainal, O. Akinsola, K. Rajamani

**Affiliations:** Rochester Regional Health/Unity Hospital, USA

## Abstract

Cushing syndrome (CS) is a disorder characterized by a result of chronic exposure to excessive glucocorticoids. This article describes a case of a 30-year-old female presenting with fatigue, abdominal striae, unintentional weight gain, and lipodystrophy. A rare diagnosis of ectopic adrenocorticotropic-dependent CS was determined and a neuroendocrine lung tumor (NET) was discovered on chest x-ray. After surgical resection, pathology confirmed lung NET that stained positive for adrenocorticotropic hormone (ACTH). The patient's symptoms fully resolved. The authors aim to urge clinicians to maintain a high index of suspicion for ectopic ACTH secretion (EAS) through a multimodal approach when caring for patients with CS.

## 1. Background

Adrenocorticotropic hormone (ACTH) overproduction leads to elevated levels of cortisol and subsequently causes Cushing syndrome (CS). CS is a constellation of symptoms and signs including central obesity, abdominal striae, round face, lipodystrophy, fatigue, hypertension, myopathy, osteoporosis, acne, hirsutism, and oligomenorrhea or amenorrhea [1]. Cognitive dysfunction including depression and memory loss can also occur. The most common cause is iatrogenic from glucocorticoid therapy [1]. Another cause is ACTH-independent from an adrenal gland tumor producing cortisol. The second most common cause is ACTH-dependent CS secondary to ACTH-secreting pituitary adenoma, which stimulates cortisol release from the adrenal glands [1]. This is referred to as Cushing disease. ACTH-dependent CS from ectopic ACTH secretion (EAS) accounts for only 10% of those cases [1, 2]. Locating the ACTH source and treating patients can be incredibly challenging.

Investigations including elevated morning glucose, serum cortisol, 24-hour urine cortisol, and midnight plasma cortisol can aid in the diagnosis of CS. Low dose dexamethasone suppression testing failing to suppress endogenous cortisol secretion is confirmatory. A high dose dexamethasone suppression test is more likely to exert negative feedback on ACTH-producing pituitary cells but less likely with ectopic ACTH-producing cells or adrenal cells. Discriminating between EAS and CD can be convoluted. Misdiagnosis can occur due to discordant imaging and biochemical investigations. Current guidelines recommend that inferior petrosal sinus sampling (IPSS) is the gold standard in distinguishing CD from EAS if pituitary MRI is negative [1, 2]. IPSS is limited by the expertise required, its invasive nature, high cost, and risk for thromboembolic complications. As such, utilizing clues from lab investigations can help physicians to consider EAS and promptly search for ectopic sources. Pulmonary neuroendocrine tumors tend to be occult, less likely to metastasize, and carry a good prognosis when diagnosed in a timely manner [2]. We herein report a rare case of ectopic ACTH producing neuroendocrine lung tumor causing CS.

## 2. Case Presentation

A 30-year-old Vietnamese female presented to the clinic with fatigue, muscle weakness, memory loss, unintentional weight gain, hair loss, and amenorrhea. She has a past medical history of microprolactinoma discovered on recent pituitary magnetic resonance imaging (MRI) treated with cabergoline. A follow-up pituitary MRI was normal and her cabergoline was discontinued. She is not taking any medications and has no known allergies. She has a family history of diabetes and coronary artery disease. She has not had any previous surgeries.

**Figure 1:**
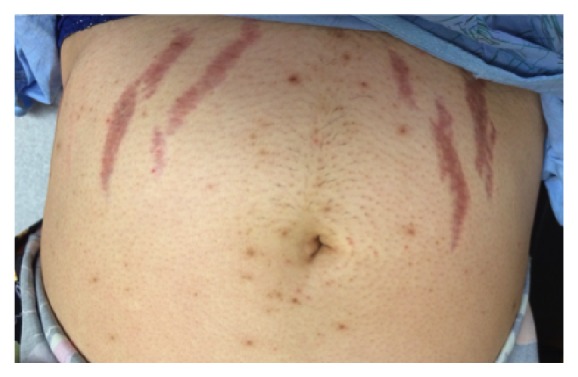
Abdominal striae.

On physical examination, her blood pressure was 132/80 and other vital signs were normal. She had facial acne, hirsutism, round face, lipodystrophy, central obesity, and muscle wasting in her lower extremities. She had no evidence of thyromegaly. Auscultation revealed normal heart sounds and no murmurs. Lung auscultation revealed normal breath sounds. Her abdomen was distended, nontender, with thick purple striae (Figure 1). She had ecchymosis and upper extremity striae.

Her morning cortisol was 53.6 (4.3-22.4mcg/dl) and ACTH was 147 (0-46pg/ml). Her midnight salivary cortisol level was 2.54 (<0.09mcg/dL) and urine 24-hour cortisol was 4405 (4-55mcg/24h). Corticotropin releasing factor was 1.9 (<10pg/ml). She had a white cell count of 13 (4.0-10.0 x10^9^) and TSH 0.05 (0.55-4.78 mIU/L) with normal free T4 1.1 (0.9-1.8ng/dl), likely secondary to excessive glucocorticoid levels or sick euthyroid syndrome. Her serum potassium was 3.3 (3.5-4.5mmol/L). Her glycosylated hemoglobin was 6.4% and her insulin-like growth factor was normal. Prolactin, luteinizing hormone, and follicular stimulating hormone levels were all normal. Other labs including electrolytes, renal function, and blood counts were normal. A low-dose 1mg and a high-dose 8mg dexamethasone suppression test failed to suppress cortisol, raising our suspicion for EAS. Surprisingly, upon review of her brain magnetic resonance imaging (MRI) from Vietnam at the time of diagnosis and posttreatment with cabergoline by our neuroradiologist, this did not reveal a microprolactinoma indicating misdiagnosis. The patient's screening for multiple endocrine neoplasia (MEN) syndrome was negative.

**Figure 2:**
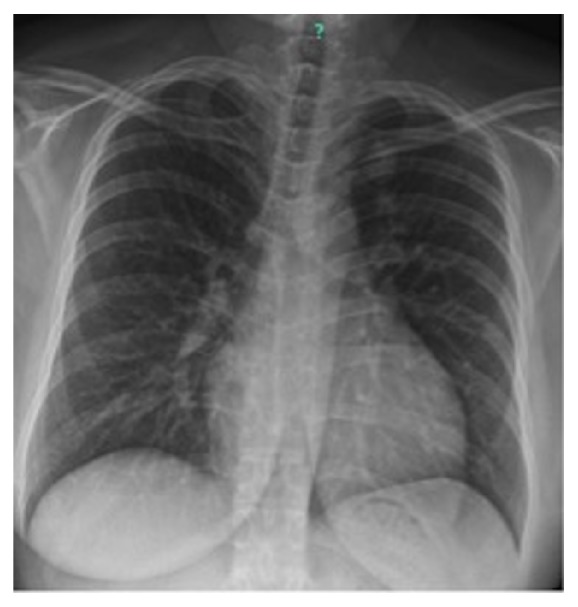
Chest X-Ray showing 2 left upper lobe lung masses.

**Figure 3:**
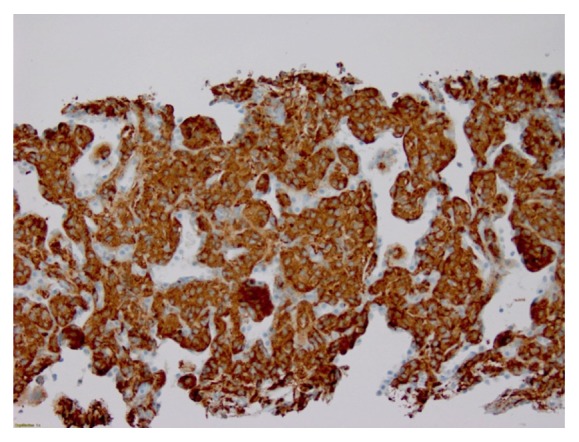
Neuroendocrine lung tumor biopsy staining positive for synaptophysin.

**Figure 4:**
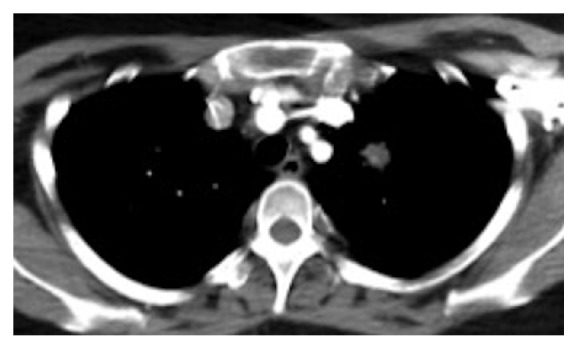
Commuted tomography showing 2 left upper lobe lung masses.

**Figure 5:**
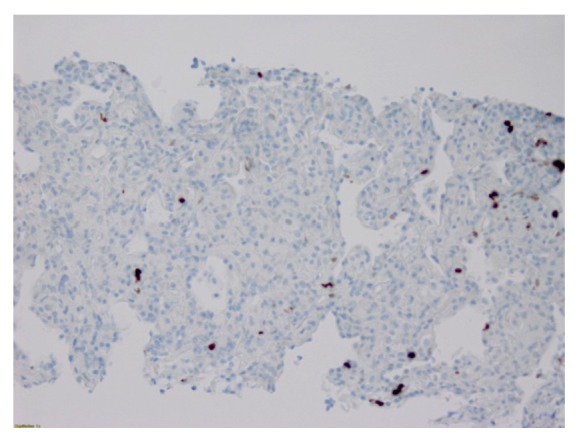
Neuroendocrine lung tumor biopsy with <2% Ki-67 proliferation index.

**Figure 6:**
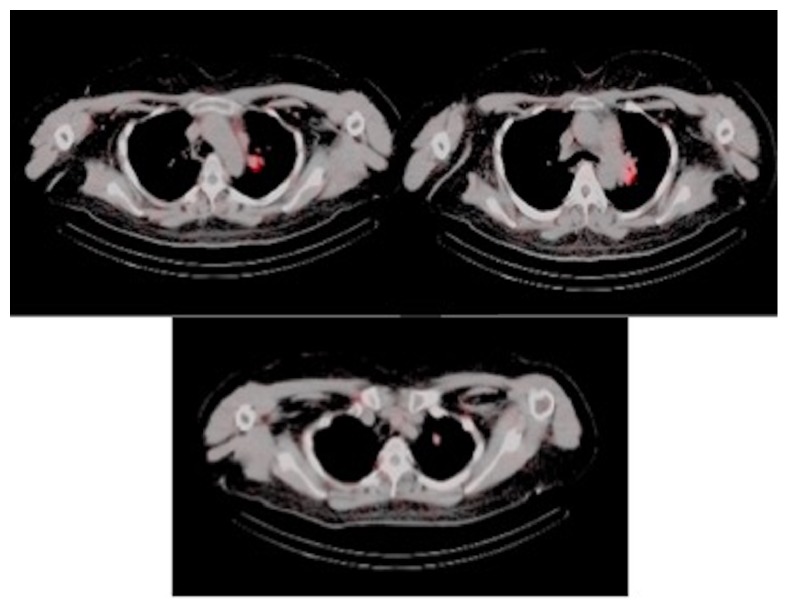
Positron emission tomography/commuted tomography showing 2 left upper lobe lung masses.

**Figure 7:**
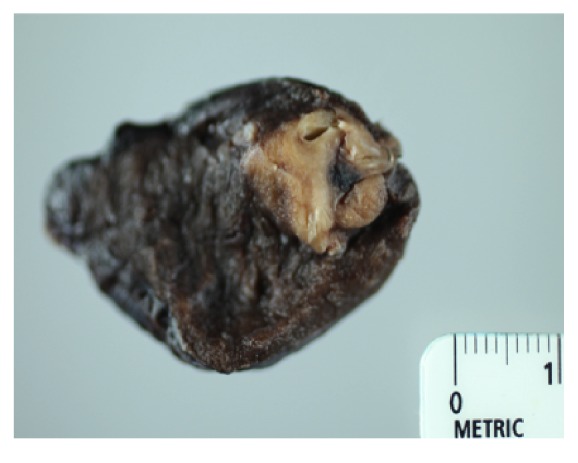
Surgical specimen of one of the resected lung masses.

Given our high clinical suspicion for EAS, we performed a chest x-ray, which identified two left upper lobe lung masses (Figure 2). A computed tomography (CT) guided lung biopsy confirmed well-differentiated NET (Figure 3). The lung biopsy stained positive for synaptophysin, chromogranin, and pancytokeratin (Figure 4). The Ki-67 proliferation index was low <2% (Figure 5). Positron emission tomography showed hypermetabolic activity within the lung masses, left hilum, and mediastinal lymph nodes (Figure 6). Hypermetabolic activity in the right anterior second rib was evident raising concern for a fracture. Mild metabolic activity in the medial limb of the left adrenal gland was present, possibly showing evidence of a small adenoma. DEXA scan was normal, but her spontaneous rib fracture confirmed a diagnosis of osteoporosis as a result of excess endogenous glucocorticoid production. Octreotide scan showed increased radiotracer uptake within the lung masses and corresponding lymph nodes. She underwent wedge resection of her left upper lobe and was treated with a steroid taper perioperatively (Figure 7). The patient's surgical margins were clear. She required intermittent insulin therapy for hyperglycemia.

## 3. Outcome and Follow-Up

After surgical resection of the neuroendocrine lung tumor, her labs normalized. Her ACTH levels were 40.9 (0-46pg/ml) and morning cortisol was 10.2 (4.3-22.4mcg/dl). Her glucose levels normalized and her insulin was discontinued. She lost 8 pounds and gained more energy. Her cognitive dysfunction resolved and her menstrual cycles regulated. She remained asymptomatic at a 6-month follow-up visit.

## 4. Discussion

Endogenous CS is uncommon with an incidence of 0.7-2.4 cases per million per year [1]. Differentiating between ACTH-dependent CS secondary to pituitary and ectopic ACTH production can be exceptionally complex.

10% of ACTH-dependent CS can be overly sensitive to dexamethasone suppression testing exhibiting false negative results [3]. 31% of patients with EAS that respond appropriately to dexamethasone suppression have pulmonary carcinoids [2]. Hypokalemia is found in 10% of patients with CD and 90% of patients with EAS due to mineralocorticoid receptor activation as seen in our patient [2, 3]. EAS must be suspected if a pituitary adenoma is not visible on MRI; however, up to 40% may not have a visualizable adenoma [2, 4]. On the other hand, 10% can have incidental pituitary tumors unrelated to CS [3]. Although IPSS carries a sensitivity and specificity of 96% and 100%, respectively, and per guidelines should be the next step, it is invasive and operator dependent [3]. When IPSS is negative, EAS is suspected and initial screening for bronchopulmonary malignancies with CT scan of the chest/mediastinum is recommended. Studies have shown that pulmonary NETs are up to 4 cm in size, which prompted us to commence with a chest x-ray instead [2].

Lung neuroendocrine tumors (NETs) represent a rare 1.2% of all lung malignancies [2]. The surveillance, epidemiology, and end result (SEER) database is the largest case-series looking at cases of CS secondary to ectopic ACTH secretion over a 20-year duration. 90 patients were identified, of which 35 had neuroendocrine lung tumors [2]. Their annual incidence has climbed over the last 50 years to 1.49 per 100,000 persons [2]. They are more common in the fifth and sixth decades of life unlike our younger patient. The incidence of lung NETs is not affected by environmental factors such as tobacco and radiation exposure [2]. Biochemical findings in those patients showed potassium levels as low as 2.0 (ranging from 3.3 to 5.1meq/L) and average urine cortisol levels at 3189 (24-108 mcg/24hr) and serum ACTH 205 (9-52pg/ml) [2]. 17 cases were localized, 17 had lymphadenopathy, and only 1 case was metastatic. Approximately 50% of localized cases were curative. Lung NETs are mostly considered to have low-grade malignancy with good prognosis and rarely metastasize [2, 5].

Somatostatin receptor-based imaging is the functional imaging of choice in the workup of NETs and it is based upon the binding of a radiolabeled ligand to the somatostatin receptor (SSR).

Haug et al. made the first study that evaluated the role of ^68^Ga-DOTATATE PET/CT in suspected neuroendocrine tumors and correlated it with gold standard pathology [6]. ^68^Ga-DOTATATE PET/CT was found to have a sensitivity of 81% and specificity of 90%. In another study, Haug et al. investigated the role of ^68^Ga-DOTATATE PET/CT in detection of metastatic lesions in patients with neuroendocrine cancer and compared it to gold standard pathology [7]. ^68^Ga-DOTATATE PET/CT was found to have 90% sensitivity and 82% specificity.

Neuroendocrine cancers are slow growing and ^18^F-FDG PET/CT is not commonly used for initial evaluation of the neuroendocrine cancers. Due to slow metabolic activity of the NETs in initial stages, they are not well visualized on ^18^F-FDG PET/CT but rather they demonstrate high uptake with ^68^Ga-DOTATATE because neuroendocrine tumors express significant SSR-2. In high grade NETs, when the tumor becomes poorly differentiated the uptake pattern changes to low ^68^Ga-DOTATATE uptake due to less expression of SSR-2 and high ^18^F-FDG PET/CT uptake. Kayani et al. reported a sensitivity of 82% for ^68^Ga-DOTATATE PET/CT and 66% for ^18^F-FDG PET/CT.

Interestingly, the etiology of pulmonary NETs remains largely unknown. In the majority of cases, they are sporadic in nature. MEN-1 mutations have been implicated in 5% of lung NETs [1, 2]. The majority of MEN-1 tumors have a loss of heterozygosity (LOH) of chromosome 11q13 [2]. LOH involving chromosomes 3p, 5q21, 9p, 13q13, and 7p13 have also been reported. The accurate diagnosis and characterization of CS requires a multifaceted diagnostic approach to facilitate timely and appropriate treatment.

## 5. Learning Points/Take Home Messages

- EAS accounts for 10% of ACTH-dependent CS.
- Diagnosing EAS can be challenging and a high index of clinical suspicion is required if high dose dexamethasone 8mg fails to suppress ACTH/cortisol secretion.
- 90% of EAS will have hypokalemia.
- Pulmonary NETs are up to 4cm in size.
- MEN-1 mutations have been implicated in 5% of lung NETs.
